# Comparison of Artificial Intelligence–Derived Heart Age with Chronological Age Using Normal Sinus Electrocardiograms in Patients with No Evidence of Cardiac Disease

**DOI:** 10.3390/jcm14155548

**Published:** 2025-08-06

**Authors:** Myoung Jung Kim, Sung-Hee Song, Young Jun Park, Young-Hyun Lee, Jongwoo Kim, JaeHu Jeon, KyungChang Woo, Juwon Kim, Ju Youn Kim, Seung-Jung Park, Young Keun On, Kyoung-Min Park

**Affiliations:** 1 Division of Cardiology, Department of Internal Medicine, Heart Vascular Stroke Institute, Samsung Medical Center, Sungkyunkwan University School of Medicine, Seoul 06351, Republic of Korea; 2 Wellysis Corp., Seoul 06133, Republic of Korea; 3 Division of Cardiology, Department of Internal Medicine, Wonju Severance Christian Hospital, Yonsei University Wonju College of Medicine, Wonju 26426, Republic of Korea; 4 Sungkyunkwan University School of Medicine, Seoul 16419, Republic of Korea; 5 MediFarmSoft Co., Ltd., Seoul 05836, Republic of Korea

**Keywords:** artificial intelligence, electrocardiogram, chronological age, biological age

## Abstract

**Background/Objectives**: Chronological age (CA) is commonly used in clinical decision-making, yet it may not accurately reflect biological aging. Recent advances in artificial intelligence (AI) allow estimation of electrocardiogram (ECG)-derived heart age, which may serve as a non-invasive biomarker for physiological aging. This study aimed to develop and validate a deep learning model to predict ECG-heart age in individuals with no structural heart disease. **Methods**: We trained a convolutional neural network (DenseNet-121) using 12-lead ECGs from 292,484 individuals (mean age: 51.4 ± 13.8 years; 42.3% male) without significant cardiac disease. Exclusion criteria included missing age data, age <18 or >90 years, and structural abnormalities. CA was used as the target variable. Model performance was evaluated using the coefficient of determination (R^2^), Pearson correlation coefficient (PCC), mean absolute error (MAE), and root mean square error (RMSE). External validation was conducted using 1191 independent ECGs. **Results**: The model demonstrated strong predictive performance (R^2^ = 0.783, PCC = 0.885, MAE = 5.023 years, RMSE = 6.389 years). ECG-heart age tended to be overestimated in younger adults (≤30 years) and underestimated in older adults (≥70 years). External validation showed consistent performance (R^2^ = 0.703, PCC = 0.846, MAE = 5.582 years, RMSE = 7.316 years). **Conclusions**: The proposed AI-based model accurately estimates ECG-heart age in individuals with structurally normal hearts. ECG-derived heart age may serve as a reliable biomarker of biological aging and support future risk stratification strategies.

## 1. Introduction

Aging is a fundamental determinant of health and disease. Although chronological age (CA) is a crucial factor in clinical decision-making, it does not always accurately reflect an individual’s true physiological condition [1]. Therefore, biological age (BA), which is influenced by genetic, environmental, and lifestyle factors, has been proposed as a more precise indicator of health status and disease susceptibility [2,3]. Accurate estimation of BA can provide a better understanding of an individual’s aging trajectory and help predict disease risk, longevity, and overall health outcomes [3,4]. As the demand for reliable biomarkers of aging grows, artificial intelligence (AI) has emerged as a promising tool for enhancing age prediction beyond conventional methods.

Recent advances in AI have enabled the development of models that estimate ECG (electrocardiogram)-derived heart age, a novel biomarker of BA based on ECG patterns. As a measure of BA, ECG-heart age captures age-related changes in cardiac electrophysiology, reflecting both structural and functional alterations beyond CA. These models use deep learning algorithms to analyze standard 12-lead ECGs, capturing subtle electrical variations associated with aging and cardiovascular remodeling [5,6]. Several studies have demonstrated that ECG-heart age correlates strongly with cardiovascular risk factors, systemic aging, and all-cause mortality, suggesting its potential role in early disease detection, risk stratification, and personalized health monitoring [1,4,7]. Compared with traditional biomarkers of aging, AI-driven ECG-heart age estimation is a non-invasive, widely accessible, and cost-effective method to assess BA at both individual and population levels [3].

Despite its potential, the clinical validity and reliability of ECG-heart age, compared with CA, remains an area of active investigation. Existing studies suggest that ECG-based AI models can effectively predict cardiovascular outcomes, but their accuracy across diverse populations and different age groups has yet to be fully elucidated [8]. Additionally, potential biases in AI-predicted ECG-heart age estimation and the implications of those biases for clinical decision-making require further evaluation. This study aimed to assess the utility of AI-derived ECG-heart age, predicted using a convolutional neural network (CNN), as a proxy for biological aging by analyzing individuals without evidence of structural heart disease. By minimizing disease-related confounding, we sought to isolate physiological aging signals embedded in the ECG and evaluate their relevance for future clinical risk prediction.

## 2. Materials and Methods

### 2.1. Study Population

This study was conducted using ECG data from patients who visited a single tertiary institute in the Republic of Korea between January 2010 and January 2024, under the approval of the Institutional Review Board (IRB No. 2020-01-007). Data collection was conducted according to the principles outlined in the Declaration of Helsinki (2013) and Good Clinical Practices. Informed consent was waived by the IRB due to the retrospective design of the study. Initially, 2,065,124 ECGs were available for analysis. To ensure data quality and relevance, patients with missing age information, those younger than 18 years or older than 90 years, and those with significant cardiac disease conditions were excluded. The specific significant cardiac conditions excluded were aborted sudden cardiac death, cardioverter-defibrillator (ICD) or cardiac resynchronization therapy (CRT) implantation before ECG acquisition, and atrial fibrillation (AF). For coronary artery disease (CAD), individuals with stenosis of ≥50% on coronary angiography or coronary computed tomography or a history of revascularization were excluded, whereas those with stenosis of <50% without prior interventions were retained to ensure a representative real-world population. For valvular heart disease, individuals with moderate to severe valvular heart disease were excluded. Patients with a left ventricular ejection fraction (LVEF) <50% on echocardiography or those diagnosed with cardiomyopathy were also excluded. Additionally, unmatched ECGs lacking patient records, diagnoses, or hospital identifiers and ECGs containing duplicate or corrupted signals were removed.

After applying those exclusion criteria, a final dataset of 292,484 ECGs was used for model development. The dataset was randomly assigned to training, validation, and testing cohorts, with no individual having ECG recordings in multiple cohorts. The test set comprised 39,990 ECGs, which were used to evaluate model performance.

### 2.2. AI Model Development and Training

**Figure 1:**
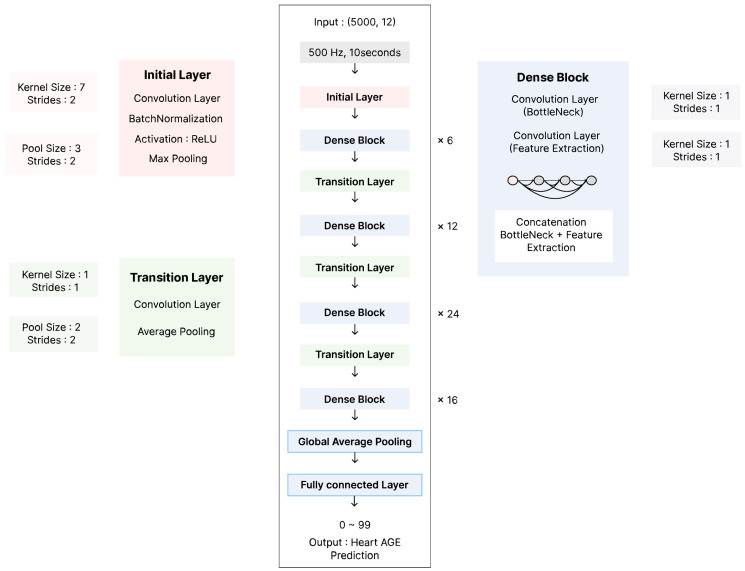
Model Architecture.

A deep learning model based on DenseNet-121, a CNN, was implemented to predict BA from standard 12-lead ECG recordings. DenseNet-121 was selected for its ability to efficiently extract hierarchical features while maintaining relatively low model complexity. The model was trained in a supervised learning framework with CA as the continuous outcome variable in a regression setting. Figure 1 illustrates the architecture of the implemented model.

The ECG signals, sampled at 500 Hz over a 10-s window, pass through an initial layer, which consists of a convolutional layer with batch normalization and ReLU activation, followed by max pooling. The extracted features are then processed through multiple dense blocks and transition layers. Each dense block consists of a bottleneck convolutional layer and multiple convolutional layers for feature extraction, with concatenation operations ensuring efficient information flow. Transition layers include convolutional layers and average pooling to progressively reduce the feature map dimensions. The final representations are aggregated using global average pooling, and the model outputs a continuous age prediction (ranging from 0 to 99) through a fully connected layer.

All ECG signals underwent a multi-step preprocessing pipeline to improve signal quality and reduce noise-related artifacts. Baseline wander was corrected using a high-pass filter with a cutoff frequency of 0.5 Hz. Powerline interference (50 Hz) was attenuated using notch filtering. All recordings were acquired using the same device at a uniform sampling rate of 500 Hz, and, therefore, resampling was not required. Signals were normalized to ensure amplitude consistency across samples. Recordings with missing leads or excessive noise were visually screened and excluded. These steps were applied uniformly to both training and validation datasets to ensure data quality and minimize preprocessing-related bias.

Prior to training, all ECG recordings were normalized and resampled to a uniform length to ensure consistency in model input. The model was optimized using the mean squared error loss function, with the Adam optimizer and a learning rate decay strategy (initial learning rate of 10 × 10^−4^) to prevent overfitting. Training was conducted with a fixed batch size of 128 over 20 epochs, incorporating early stopping based on validation loss. To enhance model generalization, data augmentation techniques, such as random noise injection and minor temporal shifting, were applied to the ECG waveforms. The model’s predictive accuracy was evaluated using standard regression performance metrics: the coefficient of determination (R^2^) to assess variance explained and the mean absolute error (MAE) and root mean squared error (RMSE) to quantify prediction accuracy. Pearson correlation coefficients (PCCs) were computed to measure the relationship between the AI-predicted heart ECG age and CA. To further assess consistency across different age groups, stratified analyses were performed, and the results were visualized using scatterplots, violin plots and confusion matrices.

### 2.3. Statistical Analysis

AI-predicted heart ECG age and CA were analyzed using descriptive statistics, with continuous variables reported as the mean ± standard deviation (SD) and categorical variables reported as numbers (percentages). To evaluate their agreement, the MAE, RMSE, R^2^ were calculated. Additionally, two-dimensional kernel density estimation with a Gaussian kernel was applied to compute density distributions, which were min-max normalized for relative comparisons. To examine trends in age differences, a locally estimated scatterplot smoothing (LOESS) regression was performed. Furthermore, violin plots incorporating box plots were constructed to visualize the distribution of age differences across age groups, and a confusion matrix was generated to assess classification agreement between the chronological and predicted age groups. For external validation, the model’s performance was evaluated using the same preprocessing and inference pipeline. R^2^, MAE, and RMSE were compared between the internal and external datasets to assess robustness across different clinical settings. All statistical analyses were conducted using R Statistical Software (version 4.2.3; R Foundation for Statistical Computing, Vienna, Austria).

## 3. Results

### 3.1. Study Population and Data Characteristics

**Figure 2:**
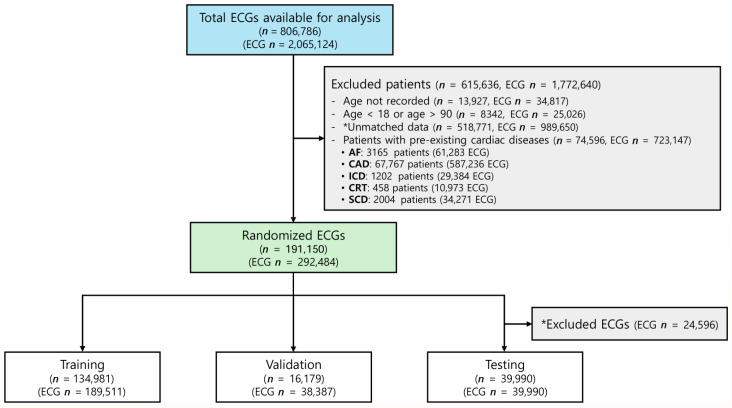
Study flow. Abbreviations: AF = atrial fibrillation, CAD = coronary artery disease with moderate or greater stenosis, ICD = patients with an implantable cardioverter defibrillator, CRT = patients with a cardiac resynchronization therapy device, SCD = patients who experienced sudden cardiac death, ECG = electrocardiogram, n = number of patients, ECG n = number of ECG recordings. *Unmatched data refer to ECGs without corresponding patient records or missing key data such as diagnosis or hospital identifiers, or those containing duplicate or corrupted signals. *Excluded ECGs refer to ECGs removed from the test set selection process to include only one ECG per patient (n = 24,596 excluded).

Initially, 2,065,124 ECGs were available for analysis. The exclusion criteria eliminated 1,772,640 ECGs, as described in the Methods section, due to missing age information (34,817 ECGs), age <18 or >90 years (25,026 ECGs), significant cardiac disease conditions (723,147 ECGs), or unmatched data (989,650 ECGs) lacking patient records, diagnoses, or hospital identifiers or containing duplicate or corrupted signals. As a result, the final dataset contained 292,484 ECGs, which were randomly assigned to the training, validation, and test sets (Figure 2). The baseline characteristics are summarized in Table 1. The age distribution of the dataset is shown in Figure S1 and Table S1. The most represented age group was 50–59 years, accounting for 29.3% of the total cohort (n = 54,729), followed by 40–49 years (22.7%, n = 42,368) and 60–69 years (18.8%, n = 35,056). Younger individuals aged 10–19 years and elderly individuals aged 80 years or older accounted for only 0.4% (n = 804) and 1.4% (n = 2590), respectively.

### 3.2. Performance of the ECG-Heart Age Prediction Model

**Figure 3:**
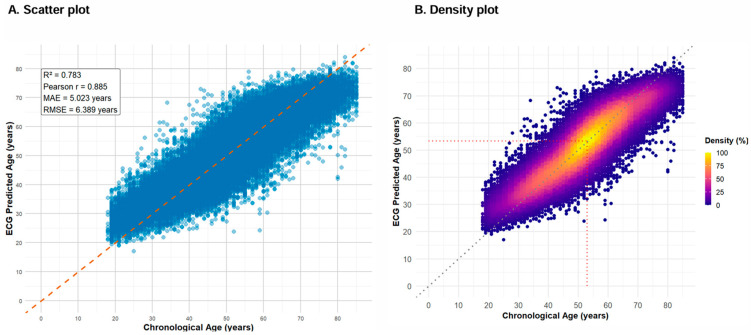
Correlation Between ECG Predicted Age and Chronological Age. (**A**) A scatter plot depicting the correlation between ECG-predicted age and chronological age. The dashed orange line represents the line of identity (y = x), indicating perfect agreement. Model performance metrics include R^2^ = 0.783, Pearson correlation coefficient (r) = 0.885, mean absolute error (MAE) = 5.023 years, and root mean square error (RMSE) = 6.389 years. (**B**) A density-colored scatter plot visualizes the distribution of predicted ECG-age. The highest density was observed at a chronological age of 53 years, where the corresponding ECG-predicted age was 53.3 years. This point is marked by the intersection of the red dotted lines, highlighting the peak density region in the plot.

The ECG-heart age prediction model achieved an R^2^ of 0.783 and a PCC of 0.885, demonstrating strong predictive performance. The mean difference between ECG-heart age and CA was −0.057 years, with an SD of 6.389 years (Table 2). It showed an MAE of 5.023 years and an RMSE of 6.389 years (Figure 3A). A density-colored scatterplot visualizes the distribution of predicted ECG-heart age (Figure 3B).

**Figure 4:**
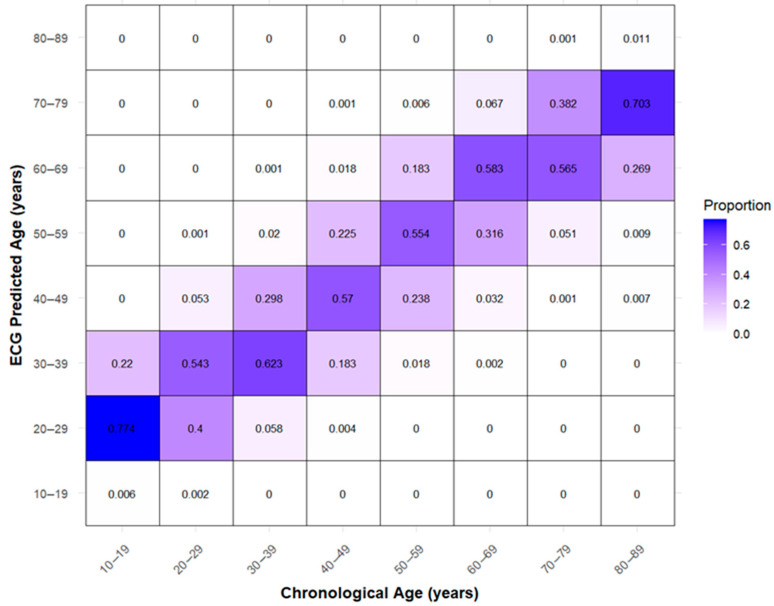
Confusion Matrix of Predicted vs. Actual Age Group.

The highest density was observed at CA 53 years, where the corresponding ECG-heart age was 53.3 years. Predictions tended to be overestimated in individuals younger than 30 years and underestimated in those older than 70 years. The confusion matrix shows systematic classification patterns across age groups (Figure 4).

Individuals in the 10–19 age group were frequently classified as 20–29, with 77.4% falling into this category, and 54.3% of those in the 20–29 group were classified as 30–39. Middle-aged individuals demonstrated relatively high classification accuracy, with 62.3% correctly classified in the 30–39 group, 57.0% in the 40–49 group, 55.4% in the 50–59 group, and 58.3% in the 60–69 group. In contrast, older individuals were often underestimated, with 56.5% of those in the 70–79 group classified as 60–69 and 70.3% of those in the 80–89 group classified as 70–79.

### 3.3. Differences Between AI-Predicted Heart Age and Actual Age

The age difference, defined as the difference between the AI-predicted heart age and CA (ECG-heart age—CA), is depicted in Figure S2 and Table 3.

**Figure 5:**
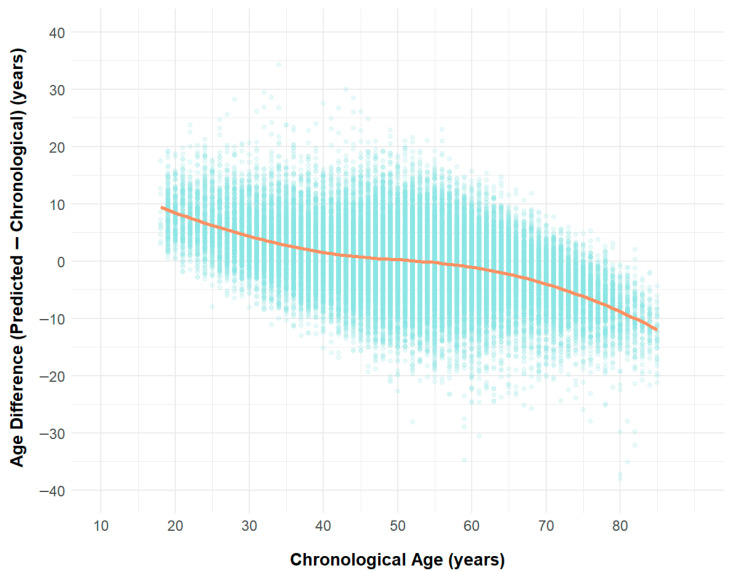
Scatter Plot of Age Difference (Predicted − Chronological) with LOESS Smoothing.

Additionally, Figure S3 and Table S2 provide an overall summary of the age differences without age stratification, with a mean of −0.057 and a median of 0.049.

### 3.4. External Validation and Model Performance

To assess the generalizability of the model, external validation was conducted using 1191 ECGs from 1191 patients at Wonju Severance Hospital. To ensure high-quality ECG inputs, each ECG was manually reviewed by a data scientist, and those showing severe baseline wander, characterized by excessive low-frequency drift across multiple leads in the multi-lead ECG signal plot, were excluded. The model achieved an R^2^ of 0.703, MAE of 5.582, and RMSE of 7.316, with a PCC of 0.846, demonstrating robust performance across different clinical settings (Table S3, Figure S4).

## 4. Discussion

ECG has played a pivotal role in cardiovascular diagnostics for more than a century, beginning with Willem Einthoven’s introduction of the string galvanometer in the early 1900s, which enabled the first precise recordings of cardiac electrical activity [9]. Einthoven’s development established ECG as a fundamental tool in cardiology. Although its clinical importance has remained consistent, the advent of echocardiography and advanced imaging modalities temporarily limited its role in aging and cardiovascular risk assessment [10,11]. However, with advances in AI and deep learning, ECG has regained importance for its ability to detect subclinical cardiovascular risk. AI-driven analyses extract latent features beyond the traditional interpretation, enhancing cardiovascular assessment and risk prediction [12].

This study demonstrates the development and validation of a CNN-based deep learning model that effectively estimates BA from standard 12-lead ECG recordings in patients with no evidence of any cardiac disease. Our model exhibited robust performance with an R^2^ of 0.783, an MAE of 5.023 years, and a PCC of 0.885, highlighting its strong predictive performance. Notably, ECG-heart age demonstrated high accuracy, particularly in middle-aged populations, while systematically overestimating age in younger individuals and underestimating it in older individuals. External validation further confirmed the model’s robustness, reinforcing its clinical applicability across diverse settings.

ECG-heart age generally aligns with CA, capturing key electrophysiological markers of aging. However, systematic discrepancies emerge at the extremes of age. Younger individuals tend to have an overestimated ECG-heart age, likely due to heightened myocardial electrical activity and autonomic influences, resulting in a mean overestimation of 8.3 years in the 10–19 group and 6.2 years in the 20–29 group. In contrast, ECG age is progressively underestimated in older adults, with a mean underestimation of 5.5 years in the 70–79 group and 10.3 years in the 80–89 group. These deviations could stem from age-related myocardial remodeling, including fibrosis, conduction slowing, and reduced autonomic variability. Additionally, selection bias might play a role because healthier individuals tend to reach advanced ages, potentially skewing ECG-heart age estimations. The predominance of middle-aged individuals in the dataset might also have contributed to higher predictive accuracy within this group.

Our findings align with previous large-scale studies of ECG-derived BA estimation. Lima et al. provided foundational evidence from a large-scale Brazilian cohort of more than 1.5 million patients, and those ECG-heart age estimations followed a similar pattern of overestimation in younger individuals and underestimation in older adults. Their findings showed that individuals whose ECG age was more than eight years greater than their CA tended to be younger on average (mean age 42.2 years), whereas those with ECG-heart ages more than eight years lower than their CA were older (mean age 63.0 years). Furthermore, they reported that a higher ECG-heart age relative to CA was associated with increased mortality risk (HR 1.79, *p* < 0.001), whereas a lower ECG-heart age corresponded to reduced mortality risk (HR 0.78, *p* < 0.001), reinforcing the prognostic relevance of ECG-heart age [13]. Building on those findings, Evans et al. investigated ECG-heart age estimation in an Australian cohort, revealing a systematic pattern across age groups. Their findings indicated that younger individuals (20–29 years) had an ECG-heart age exceeding their CA by an average of 14.3 years, whereas older individuals (80–89 years) had an ECG-heart age that was underestimated by 10.5 years [14]. Their trend aligns with our study’s observations. However, it is important to acknowledge that their study examined a hospitalized cohort with varying cardiovascular conditions, and the extent to which comorbidities influence ECG-heart age in younger patients remains unclear because they did not provide stratified disease prevalence data across age groups. Despite mounting evidence supporting ECG-derived BA estimation, inconsistencies persist across studies. For instance, Lindow et al. reported weak associations between AI-predicted ECG-heart age and cardiovascular outcomes, potentially due to variations in dataset composition, model architectures, or ECG signal preprocessing techniques [15]. Another critical challenge is the lack of transparency in how ECG inputs are transformed into clinical outputs within these models, often referred to as the “black box problem” [16]. Meanwhile, Attia et al. examined an AI-enabled ECG model for AF detection in sinus rhythm, which achieved an AUC (Area Under the Curve) of 0.87 but had an F1 score of 39.2%, reflecting challenges in balancing sensitivity and precision [17]. This suggests that although deep learning models can effectively identify ECG patterns, their diagnostic performance in real-world applications remains limited by difficulties optimizing both accuracy and clinical utility.

This study advances the field of AI-driven biological aging assessment by demonstrating the feasibility of ECG-derived heart age prediction in a large, real-world clinical cohort. This research broadens the application scope of AI-based ECG analysis to non-invasive aging assessment. Importantly, our model was developed using a cohort without significant cardiac disease, which likely contributed to the strong correlation between the ECG-derived BA and CA. Again, a major strength of this study is its use of a large, well-curated data set with stringent exclusion criteria, ensuring data reliability and robust model performance. We excluded patients with significant cardiac conditions, including AF, prior ICD or CRT implantation, and moderate-to-severe CAD, to focus on individuals without major cardiac disease. This approach minimizes the confounding effects of structural heart disease and enhances the model’s ability to assess ECG-derived BA in a relatively healthy population. External validation further enhances the credibility of these findings by confirming the model’s applicability in different clinical settings. Future studies should investigate whether this correlation remains consistent in patients with various cardiac diseases. It is essential to examine whether individuals with structural heart disease or arrhythmia exhibit discrepancies between their chronological age and AI-driven ECG age, potentially reflecting disease-related alterations in cardiac electrophysiology. ECG-derived heart age, reflecting subclinical cardiac aging, may enhance traditional risk models such as the Framingham or SCORE models when integrated into composite frameworks. Recent AI advances in arrhythmia detection, ablation planning, and risk prediction further support the role of ECG-based AI tools in personalized cardiology [18]. Emerging studies are beginning to explore such differences, and further research is warranted to determine the clinical implications of such variations.

Despite the value of this study, its limitations should be acknowledged. First, although the model demonstrated high predictive accuracy, its clinical utility in influencing therapeutic decision-making remains to be established. Second, although the exclusion of patients with significant cardiovascular conditions improved the model’s precision in a healthy population, it may limit the generalizability of the findings to individuals with established cardiac disease. Additionally, despite confirming model robustness, the limited sample size and single-center design of the external cohort may constrain the broader applicability of these results. Differences in correlation (z = 4.15, *p* < 0.001), MAE (*p* < 0.01), and RMSE (*p* < 0.01) were observed between internal and external validation, but, given limitations in the dataset and lack of raw prediction values, these findings remain exploratory. Taken together, these factors highlight that this study focused on chronological age prediction in a healthy cohort and that there remains a need for prospective studies to evaluate the prognostic value and clinical applicability of ECG-derived heart age, especially in diverse populations including those with cardiovascular disease. Third, the dataset was predominantly composed of middle-aged individuals, which might have contributed to the model’s high predictive accuracy in this group. Fourth, the inherent opacity of deep learning models limits the interpretability of ECG-heart age. Applying explainable AI techniques such as SHAP (Shapley Additive Explanations) and Grad-CAM (Gradient-weighted Class Activation Mapping) can help identify which ECG segments contribute most to the prediction. Enhancing model transparency may improve clinician trust and meet regulatory expectations, thereby supporting clinical adoption.

## 5. Conclusions

Our AI-driven ECG-heart age prediction model is a reliable and non-invasive tool for assessing biological aging in patients with normal hearts. The model demonstrated strong predictive performance, with high correlation to CA and robust validation across diverse clinical cardiac settings. Although the ECG-heart age aligned well in middle-aged individuals, systematic overestimation in younger individuals and underestimation in older adults highlight areas for further refinement.

## Figures and Tables

**Table 1:** Baseline characteristics.

Characteristic	Total (n = 191,150)
Demographics	
Age, years	51.4 ± 13.8
Male, n (%)	80,808 (42.3)
BMI (kg/m^2^)	16.7 ± 11.4
HR (bpm)	77.9 ± 13.7
Echocardiographic Measures	
LVEF (%)	64.9 ± 5.7
LVIDd (mm)	47.9 ± 4.5
LVIDs (mm)	28.3 ± 3.6
IVSd (mm)	8.6 ± 1.2
LAVI (mL/m^2^)	29.3 ± 8.0
E/A ratio	1.0 ± 0.4
E/e′ ratio	8.1 ± 2.5
RAP (mmHg)	5.2 ± 1.1
RVSP (mmHg)	26.4 ± 7.3
LVMI (g/m^2^)	85.0 ± 21.0
Laboratory Data	
NT-proBNP (pg/mL)	88.3 ± 110.1
Medical History	
DM, n (%)	7352 (3.8)
HTN, n (%)	21,948 (11.5)
CKD, n (%)	4359 (2.3)
CAD, n (%)	406 (0.2)
PAOD, n (%)	1228 (0.6)
CVA, n (%)	1743 (0.9)

Abbreviations: BMI = body mass index; CAD = coronary artery disease; CKD = chronic kidney disease; CVA = cerebrovascular accident; DM = diabetes mellitus; E/A ratio = ratio of early mitral inflow velocity (E) to atrial contraction velocity (A); E/e′ ratio = ratio of mitral inflow velocity (E) to mitral annular velocity (e′); HR = heart rate; HTN = hypertension; IVSd = interventricular septal wall thickness; LAVI = left atrial volume index; LVEF = left ventricular ejection fraction; LVMI = left ventricular mass index; LVIDd = left ventricular internal diameter in diastole; LVIDs = left ventricular internal diameter in systole; NT-proBNP = N-terminal pro B-type natriuretic peptide; PAOD = peripheral arterial occlusive disease; RAP = right atrial pressure; RVSP = right ventricular systolic pressure.

**Table 2:** Statistical Summary of Age Prediction Model.

R^2^	MAE	RMSE	Pearson Correlation	Difference Age Mean	Difference Age SD	n
0.783	5.023	6.389	0.885	−0.057	6.389	39,990

**Table 3:** Descriptive Statistics of Age Difference by Age Group.

	Age Difference (ECG Age − Chronological Age)							Chronological Age		ECG Age		
Age Group	Min	Max	Mean ± SD	Median	IQR	Q1 (25%)	Q3 (75%)	Mean ± SD	Median	Mean ± SD	Median	n
10–19	0.49	19.3	8.3 ± 3.96	8.5	5.32	5.34	10.7	18.9 ± 0.25	19	27.2 ± 3.98	27.5	177
20–29	−7.96	28.2	6.2 ± 4.67	5.81	6.21	2.99	9.2	25.4 ± 2.81	26	31.6 ± 4.88	31.2	2486
30–39	−13.1	34.3	2.81 ± 5.2	2.61	6.63	−0.71	5.92	35.0 ± 2.8	35	37.8 ± 5.46	37.4	5665
40–49	−21.1	30	0.69 ± 5.97	0.58	7.65	−3.22	4.43	45.0 ± 2.87	45	45.7 ± 6.45	45.4	8961
50–59	−34.7	23	−0.19 ± 6.17	−0.02	8.14	−4.17	3.97	54.4 ± 2.83	54	54.2 ± 6.61	54.3	11,569
60–69	−30.6	15.6	−2.02 ± 5.63	−1.68	7.29	−5.49	1.8	63.9 ± 2.78	64	61.8 ± 5.94	62.3	7135
70–79	−28	7.77	−5.54 ± 4.67	−5.25	6.06	−8.38	−2.32	73.7 ± 2.74	73	68.2 ± 4.75	68.7	3461
80–89	−38	1.99	−10.3 ± 4.75	−9.86	4.94	−12.4	−7.48	81.8 ± 1.58	82	71.5 ± 4.76	71.8	536

In younger individuals, ECG-heart age tended to be overestimated, with a mean difference of 8.3 ± 3.96 in the 10–19 group, 6.2 ± 4.67 in the 20–29 group, and 2.81 ± 5.2 in the 30–39 group. The discrepancy narrowed with increasing age, approaching zero in the 50–59 group (−0.19 ± 6.17). In older individuals, ECG-heart age was increasingly underestimated, with mean differences of −2.02 ± 5.63 in the 60–69 group, −5.54 ± 4.67 in the 70–79 group, and −10.3 ± 4.75 in the 80–89 group. Figure 5 visualizes these patterns with a scatterplot and LOESS smoothing, showing the trend of age difference in a continuous manner.

## Data Availability

The data that support the findings of this study are available from the corresponding author upon reasonable request.

## Supplementary Materials

The following supporting information can be downloaded at https://www.mdpi.com/article/10.3390/jcm14155548/s1: Figure S1. Age Distribution of the Modeling Dataset; Figure S2. Distribution of Age Differences Across Age Groups; Figure S3. Distribution of Age Difference; Figure S4. External Validation Results of Age Prediction Model; Table S1. Age Distribution of the Modeling Dataset; Table S2. Summary Statistics of Predicted vs. Actual Age Differences; Table S3. External Validation Results of Age Prediction Model.

## Abbreviations

The following abbreviations are used in this manuscript:

AF	Atrial Fibrillation
AI	Artificial Intelligence
BA	Biological Age
BMI	Body Mass Index
CA	Chronological Age
CAD	Coronary Artery Disease
CKD	Chronic Kidney Disease
CNN	Convolutional Neural Network
CRT	Cardiac Resynchronization Therapy
CVA	Cerebrovascular Accident
DM	Diabetes Mellitus
ECG	Electrocardiogram
HR	Heart Rate
HTN	Hypertension
ICD	Implantable Cardioverter Defibrillator
IQR	Interquartile Range
IVSd	Interventricular Septal Wall Thickness
LAVI	Left Atrial Volume Index
LOESS	Locally Estimated Scatterplot Smoothing
LVEF	Left Ventricular Ejection Fraction
LVIDd	Left Ventricular Internal Diameter in Diastole
LVIDs	Left Ventricular Internal Diameter in Systole
LVMI	Left Ventricular Mass Index
MAE	Mean Absolute Error
NT-proBNP	N-terminal pro B-type Natriuretic Peptide
PAOD	Peripheral Arterial Occlusive Disease
PCC	Pearson Correlation Coefficient
R^2^	Coefficient of Determination
RAP	Right Atrial Pressure
RMSE	Root Mean Square Error
RVSP	Right Ventricular Systolic Pressure
SD	Standard Deviation
