# Supplementary material for: Comparison of Artificial Intelligence–Derived Heart Age with Chronological Age Using Normal Sinus Electrocardiograms in Patients with No Evidence of Cardiac Disease
Source: J Clin Med. 2025 Aug 6;14(15):5548. doi: 10.3390/jcm14155548 (PMC12347350; doi:10.3390/jcm14155548)
Supplement: Supplementary file 1 [file jcm-14-05548-s001.zip › jcm-3715371-supplementary.pdf]

Journal of Clinical Medicine

Supplementary Materials:

Figure S1. Age Distribution of the Modeling Dataset

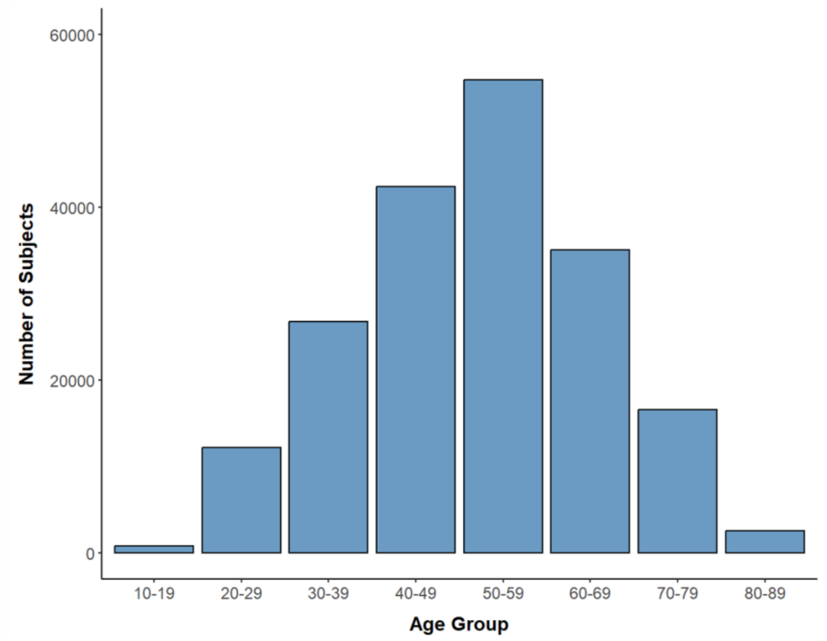

*Note: Age group is presented in years.

Figure S2. Distribution of Age Differences Across Age Groups

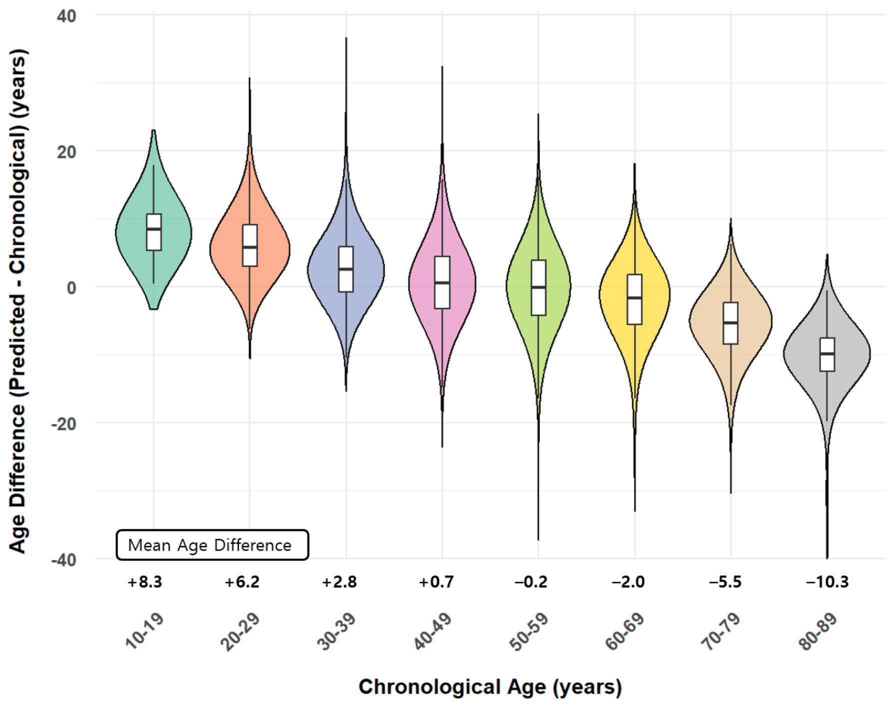

Figure S3. Distribution of Age Difference

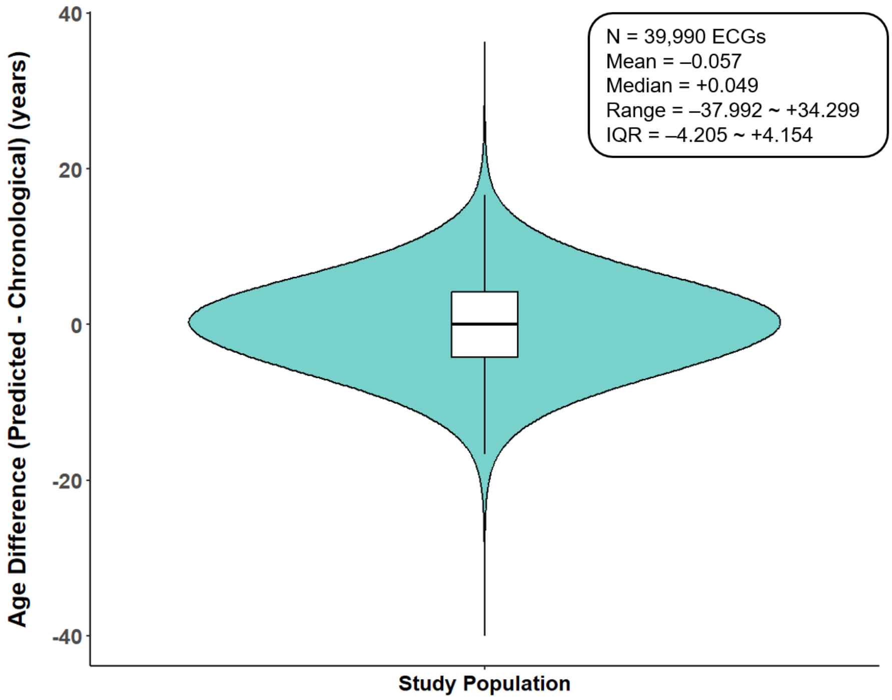

Figure S4. External Validation Results of Age Prediction Model

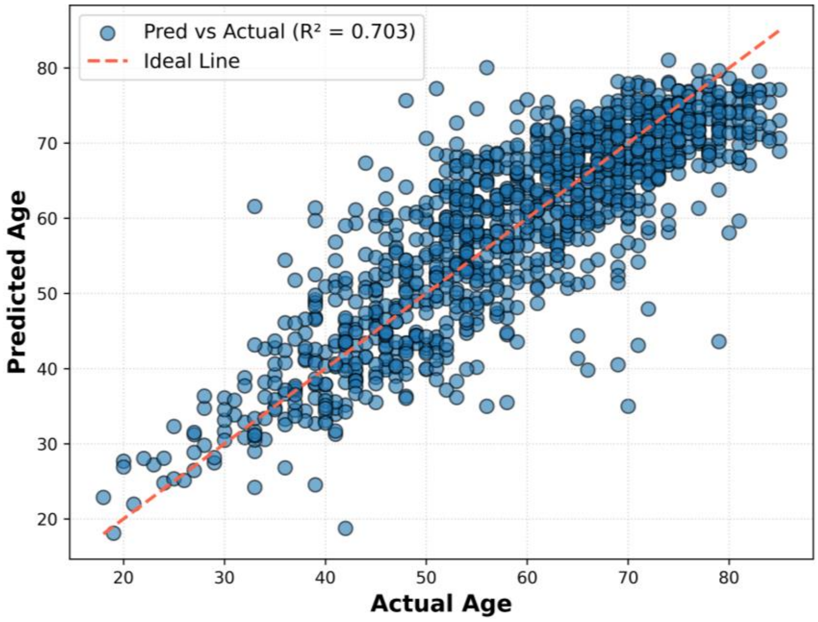

Table S1. Age Distribution of the Modeling Dataset

Age Group (years)	10–19	20–29	30–39	40–49	50–59	60–69	70–79	≥ 80
Count (n)	804	12,208	26,789	42,368	54,729	35,056	16,606	2,590

Table S2. Summary Statistics of Predicted vs. Actual Age Differences

Min	1st Qu.	Median	Mean	3rd Qu.	Max
-37.992	-4.205	0.049	-0.057	4.154	34.299

Table S3. External Validation Results of Age Prediction Model

R^2	MAE	RMSE	Pearson Correlation	Difference Age Mean	Difference Age SD	N
0.703	5.582	7.316	0.846	0.213	7.316	1,191
